# *TERT* Promoter Mutations Frequency Across Race, Sex, and Cancer Type

**DOI:** 10.1093/oncolo/oyad208

**Published:** 2023-07-18

**Authors:** Talal El Zarif, Marc Machaalani, Rashad Nawfal, Amin H Nassar, Wanling Xie, Toni K Choueiri, Mark Pomerantz

**Affiliations:** Lank Center for Genitourinary Oncology, Dana-Farber Cancer Institute, Boston, MA, USA; Department of Medicine, Harvard Medical School, Boston, MA, USA; Department of Internal Medicine, Yale New Haven Hospital, New Haven, CT, USA; Faculty of Medical Sciences, Lebanese University, Beirut, Lebanon; Faculty of Medical Sciences, Lebanese University, Beirut, Lebanon; Department of Hematology/Oncology, Yale New Haven Hospital, New Haven, CT, USA; Department of Data Sciences, Dana-Farber Cancer Institute, Boston, MA, USA; Lank Center for Genitourinary Oncology, Dana-Farber Cancer Institute, Boston, MA, USA; Department of Medicine, Harvard Medical School, Boston, MA, USA; Lank Center for Genitourinary Oncology, Dana-Farber Cancer Institute, Boston, MA, USA; Department of Medicine, Harvard Medical School, Boston, MA, USA

**Keywords:** *TERT* mutation, disparities in cancer care, immune checkpoint inhibitor

## Abstract

**Background:**

Telomerase reverse transcriptase (*TERT*) gene promoter mutations have been explored, as biomarkers of improved survival for patients with cancer receiving immune checkpoint inhibitors. We sought to investigate their prevalence by race and sex across different cancer types to inform patient selection in clinical trials.

**Results:**

In this observational study, 31 925 patients with cancer underwent next-generation sequencing of their tumors with 88% (27 970) patients self-reported being Whites, 7.1% (2273) Asians, and 5.3% (1682) Blacks. Examining the distribution of *TERT* promoter mutations by race, White patients with melanoma harbored more *TERT* promoter mutations than Asian and Black patients (OR = 25.83; 95%CI, 6.84-217.42; *P* < .001). In contrast, Asian patients with head and neck cancer (HNC) harbored more *TERT* promoter mutations compared to White patients (OR = 2.47; 95%CI, 1.39-4.37; *P* = .004). In addition, the distribution of *TERT* promoter mutations differed by sex. Males were enriched for *TERT* gene promoter mutations compared to females with melanoma (OR = 1.82; 95%CI, 1.53-2.16; *P* < .001), cancer of unknown primary (OR = 1.96; 95%CI, 1.43-2.69; *P* < .001), hepatobiliary (OR = 3.89; 95%CI, 2.65-5.69; *P* < .001), and thyroid cancers (OR = 1.42; 95%CI, 1.10-1.84; *P* = .0087), while females were more enriched for *TERT* promoter mutations compared to males for HNC (OR = 0.56; 95%CI, 0.39-0.81; *P* = .0021).

**Conclusions:**

The prevalence of *TERT* gene promoter mutations varies among patients with cancer based on race and sex. These findings inform our understanding of cancer biology and can assist in the design of future clinical trials that leverage drugs targeting *TERT* promoter dependencies.

## Teaser

*TERT* gene promoter mutations have been explored as biomarkers of improved survival for patients with cancer receiving immune checkpoint inhibitors. This article reports their prevalence by race and sex across different cancer types to inform patient selection in clinical trials.

**Implications for Practice**

The findings of this study illustrate that there are major differences in the prevalence of *TERT* promoter mutations in various cancer types across patient populations when stratifying them by sex and ethnic groups. The data presented in this work highlight the necessity of investing more resources in mutation analyses of non-White populations. These efforts will mitigate racial disparities, assist in equitable future clinical trial design, and potentially lead to better biological insights that can justify the observed differences in mutational landscapes.

## Introduction

Telomerase is a reverse transcriptase that synthesizes the 3ʹ end of linear chromosomes.^1,2^ In human somatic cells, telomeres shorten with each cellular replication, as DNA polymerase cannot fully replicate the 3ʹ end of DNA.^3,4^ Telomere shortening is a cardinal feature of the aging process, where cells undergo senescence, apoptosis, or genomic instability.^5^ Telomerase counteracts this process by adding telomeres and is inhibited in most normal human cells across different tissue types. Exceptions include continually regenerating cells (eg, stratum basalis of the skin, testes, or ovaries) and cells seeking to maintain cell proliferation and avoid senescence (ie, cancer cells).^6,7^

The telomerase reverse transcriptase (*TERT*) gene encodes the enzymatic subunit of the telomerase complex.^8^ In cancer cells, activating mutations in the *TERT* promoter region can increase *TERT* protein production and the subsequent activity of telomerase, resulting in increased telomere length.^9^ Many studies have investigated the regulation of *TERT* in healthy tissues and its aberrant expression in malignant cells.^10-13^ In addition, by promoting the epithelial-mesenchymal transition,^14^*TERT* promoter mutations can increase PD-L1 expression^15^ and thus increase response to ICI therapy. While genetic risk assessments in the clinic do not routinely test for *TERT* promoter mutations, currently, the notion is gaining traction as a strategy for helping determine prognosis.^16^ In the era of immune checkpoint inhibitors (ICIs), De Kouchkovsky et al^17^ recently suggested that the presence of *TERT* promoter mutations in urothelial cancer was a predictor of improved overall survival in a cohort of 119 patients treated with ICIs. In addition, a recent pan-cancer analysis of 10 336 patients sequenced using MSK-IMPACT showed variability in the prevalence of *TERT* mutations across cancer types and found an association between the presence of *TERT* promoter mutations and higher tumor mutational burden (TMB) and neoantigen load. The authors also noted better overall survival outcomes with anti-CTLA4 therapy, among patients with melanoma harboring *TERT* promoter mutations.^18^

Given the importance of *TERT* promoter mutations in cancer development and their potential use as a biomarker for clinical outcomes and response to treatment with ICIs, we investigated the distribution of *TERT* promoter mutations by sex and race across different solid tumors. Their prevalence across specific groups of patients can potentially guide future patient selection in clinical trials and therapeutic strategies.

## Methods

### Study Design

Patients diagnosed with 1 of the 10 solid tumors most commonly harboring *TERT* gene promoter mutations (thyroid cancer, soft-tissue sarcoma, non-small cell lung cancer (NSCLC), melanoma, hepatobiliary cancer (HPBC), head and neck cancer (HNC), glioma, colorectal cancer, cancer of unknown primary, and bladder cancer) were identified from the American Association for Cancer Research (AACR) Project Genomics Evidence Neoplasia Information Exchange (GENIE) 11.0-registry.^19^ As the bait-set coverage of the *TERT* gene was available from Dana-Farber Cancer Institute (DFCI) and Memorial Sloan Kettering Cancer Center (MSKCC), we selected patients from these 2 cancer centers for this analysis. All patients underwent next-generation sequencing of tumors with CLIA-/ISO-certified panels obtained from the respective institution from which they were recruited. Mutational analyses in this study were focused on *TERT* promoter mutations. Patients whose tumors were sequenced using DFCI OncoPanel version 1 were excluded, because it does not cover the entire *TERT* gene sequence. Methodology for tissue collection, DNA extraction and sequencing using the DFCI-ONCOPANEL, and MSK-IMPACT for DFCI and MSKCC patients, respectively, were previously described in detail.^20-23^ Additional details on methodology are provided in Supplementary material.

### Statistical Analysis

The prevalence of *TERT* promoter mutations was summarized as “*N*” and percentage by sex and race across different solid tumors. The strength of associations was quantified as the odds ratio (OR) with 95% confidence intervals (95%); 2-sided Fisher’s exact tests were applied. FDR-adjusted Q-values were also calculated to correct *P*-values using the Benjamini-Hochberg method. *P*-values < .05 were considered significant as long as they maintained a *Q*-value < .05 on multiple test corrections.

## Results

**Figure 1.**
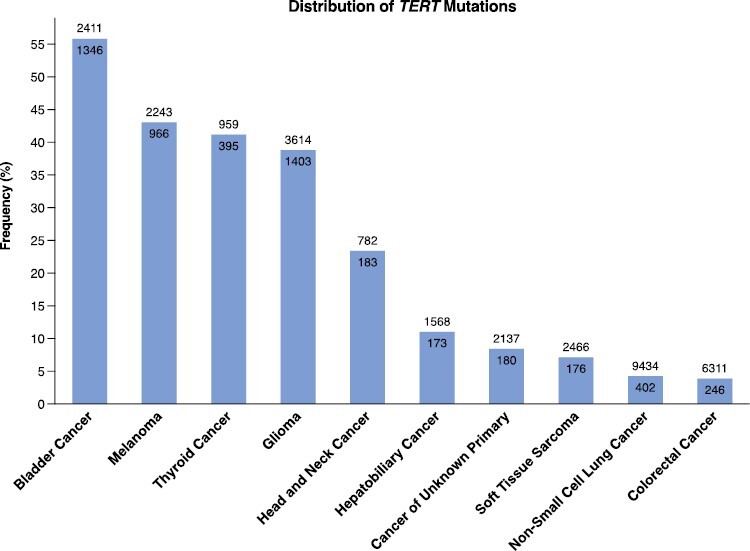
The distribution of *TERT* promoter mutations across the top 10 cancer types.

A total of 31 925 patients from DFCI and MSKCC were included (Table 1; Supplementary Table S1). In our cohort, 88% (27 970) patients self-reported being Whites, 7.1% (2273) Asians, and 5.3% (1682) Blacks. *TERT* promoter mutations were identified in 56% (1346/2411) bladder cancers, 43% (966/2243) melanomas, 39% (1403/3614) gliomas, 41% (395/959) thyroid cancers, and 23% (183/782) HNC (Fig. 1; Supplementary Table S2).

**Table 1.** Clinical and pathological characteristics of the 31 925 patients with cancer and available *TERT* sequencing data.

Patients with cancer	*n*	Percent (%)
Sex		
Female	15 350	48.1
Male	16 575	51.9
Self-reported race		
Asian	2273	7.1
Black	1682	5.3
White	27 970	87.6
Type		
Non-small cell lung cancer	9434	29.6
Colorectal cancer	6311	19.8
Glioma	3614	11.3
Soft tissue sarcoma	2466	7.7
Bladder cancer	2411	7.6
Melanoma	2243	7.0
Cancer of unknown primary	2137	6.7
Hepatobiliary cancer	1568	4.9
Thyroid cancer	959	3.0
Head and neck cancer	782	2.4

**Figure 2.**
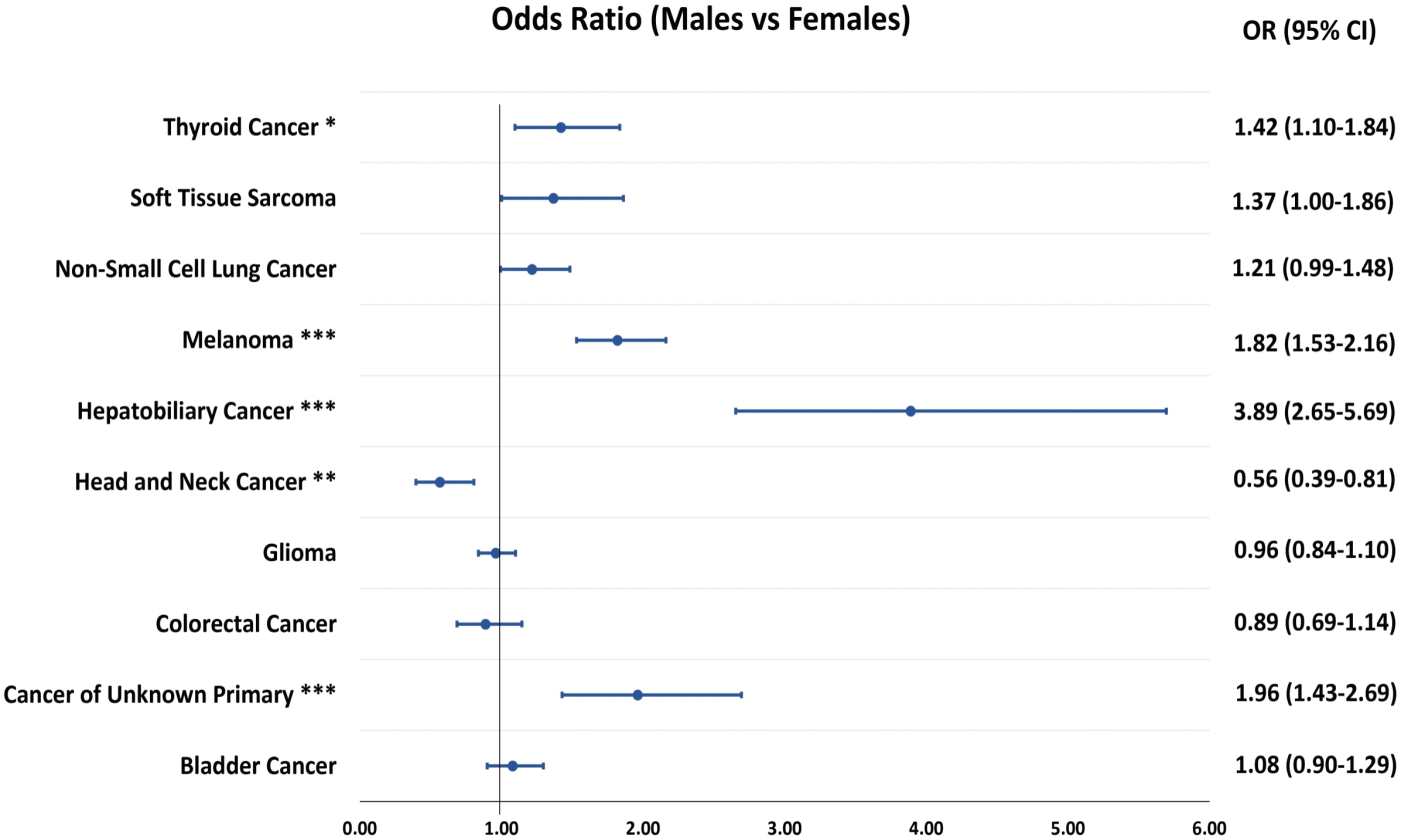
The frequency of *TERT* promoter mutations in solid tumors among males compared to females. Abbreviations: OR: Odds ratio; 95%CI: 95% confidence interval; *Q-value < 005; **Q-value < .01; ***Q-value < .001.

Examining the differences in the distribution of *TERT* promoter mutations by race, it was notable that there were differential distributions among patients with the same cancer type but belonging to different racial groups (Supplementary Tables S3 and S4). For example, White patients with melanoma (44%, *n* = 964/2176) harbored more *TERT* promoter mutations compared to Asian and Black patients (3%, *n* = 2/67) (OR = 25.83; 95%CI, 6.84-217.42; *Q* < .001) (Supplementary Table S4). Similarly, *TERT* promoter mutations were differentially enriched among patients by sex (Supplementary Table S5). *TERT* promoter mutations were significantly more frequent among male patients as compared to their female counterparts with melanoma (49%, *n* = 638/1298 vs. 35%, *n* = 328/945, respectively) (OR = 1.82; 95%CI, 1.53-2.16; *Q* < .001), HPBC (17%, *n* = 137/827 vs. 4.9%, *n* = 36/741, respectively) (OR = 3.89; 95%CI, 2.65-5.69; *Q* < .001), cancer of unknown primary (11%, *n* = 116/1065 vs. 5.9%, *n* = 64/1081, respectively) (OR = 1.96; 95%CI, 1.43-2.69; *Q* < .001), and thyroid cancer (46%, *n* = 213/468 vs. 37%, *n* = 182/491, respectively) (OR = 1.42; 95%CI, 1.10-1.84; *Q* = .017). In contrast, male patients with HNC (21%, *n* = 118/575) harbored significantly fewer *TERT* promoter mutations than female patients (31%, *n* = 65/207) (OR = 0.56; 95%CI, 0.39-0.81; *Q* = .005) (Fig. 2). When examining patients by ethnicity, there were no significant differences in the distribution of *TERT* promoter mutations between Hispanic and non-Hispanic patients (Supplementary Table S6).

**Figure 3.**
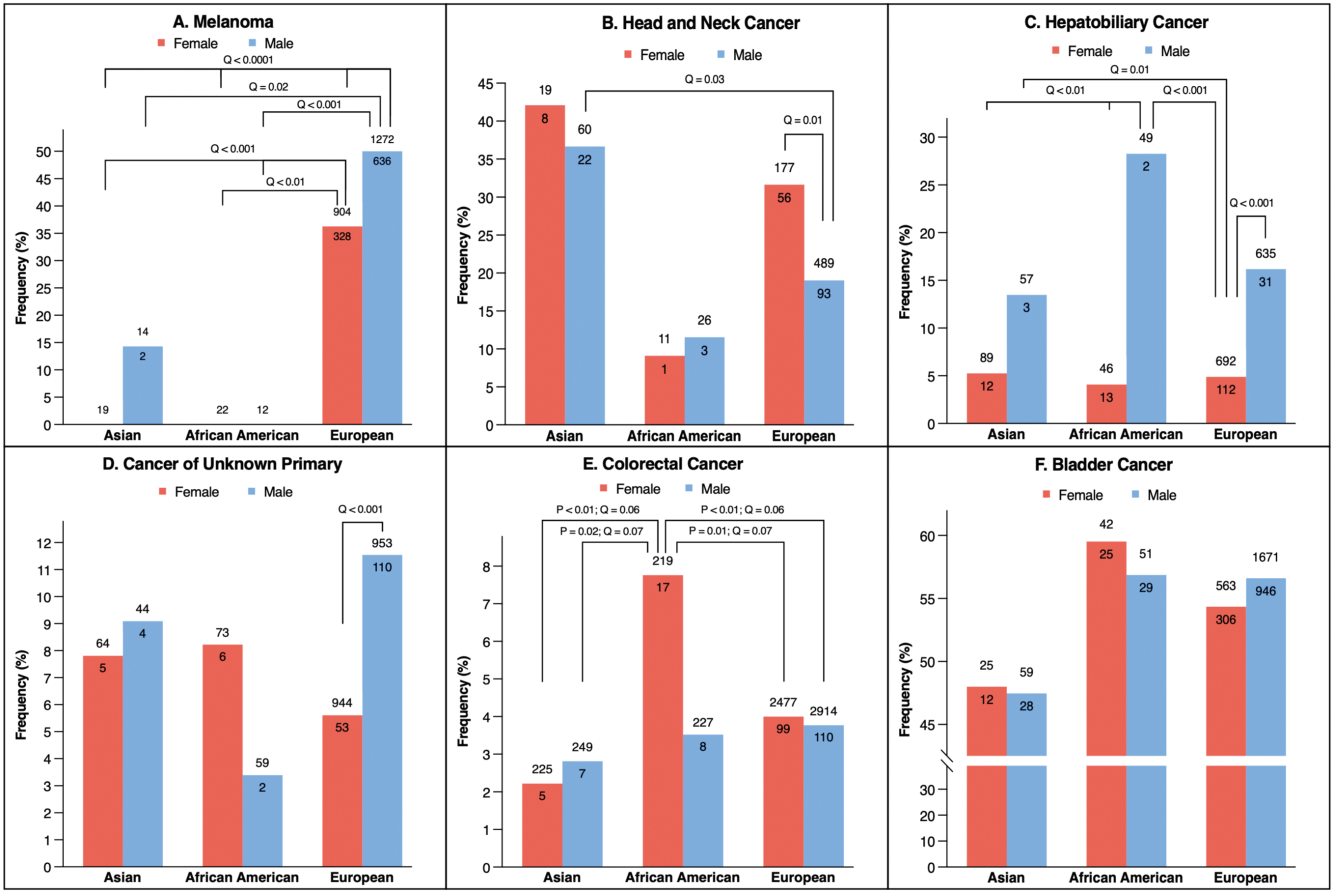
The frequency of *TERT* promoter mutations in different subgroups across cancer types.

Furthermore, *TERT* promoter mutations differed between males and females within the same race or between different racial groups in patients with the same sex. For example, *TERT* promoter mutations occurred more often in White males with HPBC (16%, *n* = 112/692) compared to their female counterparts (4.9%, *n* = 31/635) (OR = 3.76; 95%CI, 2.49-5.69; *Q* < .001), and Black males (28%, *n* = 13/46) harbored more *TERT* promoter mutations than Black females (4.1%, *n* = 2/49) (OR = 9.26; 95%CI, 1.96-43.9; *Q* = 0.008) (Fig. 3; Supplementary Table S7). Whereas in patients with HNC, Asian males (37%, *n* = 22/60) were significantly more enriched for *TERT* promoter mutations relative to White males (19%, *n* = 93/489) (OR = 2.47; 95%CI, 1.39-4.37; *P* = .004) (Fig. 3; Supplementary Table S7). No significant differences in *TERT* promoter mutations distribution were determined across racial and sex subgroups among patients with glioma, soft-tissue sarcoma, bladder, colorectal, NSCLC, and thyroid cancers (Fig. 3; Supplementary Table S7). Further exploration of HNC tumors revealed that oral cavity squamous cell carcinoma (OSCC) tumors (44%, *n* = 85/192) were associated with higher *TERT* promoter mutations vs. other HNC subtypes (17%, *n* = 98/590) (OR = 3.98; 95%CI, 2.74-5.79; *Q* = .03). Asian patients with OSCC (70%, *n* = 14/20) harbored more mutations vs. non-Asian patients with OSCC (41%, *n* = 71/172) (OR = 3.3; 95%CI, 1.2-9.0; *P* = .019) (Supplementary Table S8).

Finally, the distribution of *TERT* promoter mutations was compared between primary and metastatic tumor samples. Mutations were significantly more frequent among samples from primary tumors as compared with samples from metastatic tumors in patients with bladder cancer (58.4%, *n* = 1076/1843 vs. 38.3%, *n* = 270/559, respectively) (OR = 1.5; 95%CI, 1.2-1.8; *Q* < .001), cancer of unknown primary (12.6%, *n* = 33/261 vs. 8.2%, *n* = 133/1613, respectively) (OR = 1.6; 95%CI, 1.1-2.4; *Q* = .0248), colorectal cancer (4.5%, *n* = 191/4219 vs. 2.5%, *n* = 51/2053, respectively) (OR = 1.9, 95%CI, 1.4-2.6; *Q* < .001), glioma (42.3%, *n* = 1287/3039 vs. 25.3%, *n* = 102/403, respectively) (OR = 2.2; 95%CI, 1.7-2.7; *Q* < .001), HNC (27.4%, *n* = 116/423 vs. 19%, *n* = 67/352, respectively) (OR = 1.6; 95%CI, 1.1-2.3; *Q* = .0091), HPBC (13.3%, *n* = 148/966 vs. 5.8%, *n* = 25/433, respectively) (OR = 2.5; 95%CI, 1.6-3.9; *Q* < 0.001). However, samples from primary tumors were significantly less enriched with *TERT* promoter mutations than those from metastatic tumors in patients with melanoma (31%, *n* = 223/720 vs. 49.6%, *n* = 741/1495, respectively) (OR = 0.5; 95%CI, 0.4-0.6; *Q* < .001), soft-tissue sarcoma (6.3%, *n* = 99/1578 vs. 8.9%, *n* = 75/838, respectively) (OR = 0.7, 95%CI, 0.5-0.9; *Q* = .03), and thyroid cancer (35.2%, *n* = 177/503 vs. 48%, *n* = 217/452, respectively) (OR = 0.6; 95%CI, 0.5-0.8; *Q* < .001) (Supplementary Fig. S1; Supplementary Table S9,). The distribution of *TERT* promoter mutations were also evaluated across race and sex groups in primary (Supplementary Table S10) and metastatic (Supplementary Table S11) samples, respectively.

## Discussion

In this study, we report for the first time, differences in the distribution of *TERT* promoter mutations by race and sex across different cancer types. Our findings reveal that *TERT* promoter mutations are frequent events in many cancer types, including bladder cancer, melanoma, thyroid cancer, glioma, and HNC. *TERT* promoter mutations are found to be more prevalent among male patients than their female counterparts with most cancer types, except for HNC where the opposite held true. Additional noteworthy results include a more significant enrichment among patients of White race with melanoma and patients of Asian race with HNC, particularly OSCC.

The prevalence of *TERT* promoter mutations in melanoma varies between 29% and 71%.^24,25^ In our cohort, 43% of 2243 melanoma cases harbored *TERT* promoter mutations. A higher frequency of *TERT* mutations among male patients in melanoma compared with their female counterparts has been described.^26^ In addition, our findings reveal ethnic differences in this disease, with patients of White race carrying significantly more *TERT* promoter mutations compared to other groups. Variations in skin color and the protective effects of darker skin against ultraviolet radiation may counterpart some of this difference.^27^ Griewank et al^28^ found a considerably higher frequency of *TERT* promoter mutations in cutaneous melanomas of UV-exposure-prone areas than in tumors arising in areas with minimal or absent sun exposure.

Other factors may be implicated in these differences based on race. For example, *TERT* promoter mutations have been associated with increased patient age at diagnosis, as well as increased Breslow thickness, tumor ulceration, and tumor growth rate in patients with melanoma.^28-31^ In keeping with this, more adverse outcomes and worse survival have been consistently reported among patients with melanoma who harbor *TERT* promoter mutations compared to those who do not.^28,32,33^ However, it is notable that Black patients have a higher prevalence of advanced cutaneous melanoma^34^ with worse pathologic features (ie, tumor ulceration and satellite nodules) and regional and distant metastases.^35-37^ Rouhani et al^38^ demonstrated that Black patients presented with a significantly later stage at their time of diagnosis with melanoma compared with White American patients. As such, Black patients might also have worse outcomes due to underlying health disparities and access to care that may have led to delays in diagnosis and treatment compared to groups of patients who have more access to screening, such as skin examinations.^39^ This may explain why White patients continue to demonstrate better overall survival than non-White groups across different melanoma subtypes, irrespective of *TERT* promoter mutations.^34,36,40^

Regarding HNC, Yu et al^41^ found a mutation rate of 81% in 74 patients with OSCC. *TERT* promoter mutations were also found to be associated with an increased risk of locoregional failure (subdistribution HR = 2.82; 95%CI, 1.47-5.42; *P* = .0019) independent of oral cavity primary site and TP53 mutation status. Our study included a larger sample size and found a mutation rate of 44.27% of 192 OSCC with a frequency of 70% in OSCC in Asian patients (*n* = 20).

While certain cancer types (ie, HNC and HPBC) exhibited a higher rate of *TERT* promoter mutations in primary tumor samples compared to metastatic samples, mutations were more prevalent in metastatic samples of melanoma and thyroid cancer compared to primary tumors. Prior work by Morris et al^42^ showed that metastatic HNC tumors demonstrated a higher prevalence of *TERT* promoter mutations compared to primary tumors. Whereas Hugdahl et al^43^ found no significant difference in mutation distribution between primary and metastatic melanoma tumors. In addition, Yang et al^44^ showed that *TERT* mutant thyroid cancers were linked to a higher occurrence of distant metastasis at the time of diagnosis. Given that primary and metastatic samples may have different genomic alterations, it is imperative that future studies highlight the development of *TERT* promoter mutations throughout the course of tumor development and its progression to further understand the relationship of these mutations with tumor aggressiveness and spread.

Overall, our results add to the growing body of the literature that serves to enhance our understanding of the variation in *TERT* promoter mutations across cancer types, sex, and race. Our study has several limitations, as it included patients from tertiary academic centers who may be enriched for unique features based on referral or geographic patterns. In addition, the analyses performed relied on subjective self-reported race, rather than objective parameters (ie, ancestry). However, ancestry-based approaches have similarly found differences in the rate of *TERT* promoter mutations across ancestral groups. Arora et al^45^ recently demonstrated that patients with glioblastoma multiforme and hepatocellular carcinoma with east Asian ancestry had lower frequency of *TERT* promoter mutations compared to patients with European ancestry. Moreover, data on TMB were not available, and we were unable to adjust for it when examining the incidence of *TERT* promoter mutations across subgroups. Patients with high TMB are more likely to display genomic alterations,^46^ which may have affected their distribution across different groups. It is also noteworthy to highlight that the reporting of *TERT* promoter mutations may be significantly influenced by intratumoral heterogeneity especially when examining differences between localized tumor and metastatic specimens. Finally, heterogeneity in tumor subtypes which may have not been fully reported (ie, melanoma) may have introduced bias into our subgroups and subsequent analyses.

In conclusion, our study demonstrates distinct differences in their distribution by sex and race across several cancer types. It will be essential to account for these variations when launching future trials to assess the impact of these mutations on clinical outcomes and their use as a biomarker.

## Supplementary Material

oyad208_suppl_Supplementary_Figure_1

oyad208_suppl_Supplementary_Methods

oyad208_suppl_Supplementary_Tables

## Data Availability

The data underlying this article are available in the article and in Supplementary material.
