# Supplementary figures and images for: TERT Promoter Mutations Frequency Across Race, Sex, and Cancer Type
Source: Oncologist. 2023 Jul 18;29(1):8–14. doi: 10.1093/oncolo/oyad208 (PMC10769781; doi:10.1093/oncolo/oyad208)

# Odds Ratio (Primary vs Metastasis)

OR (95% CI)

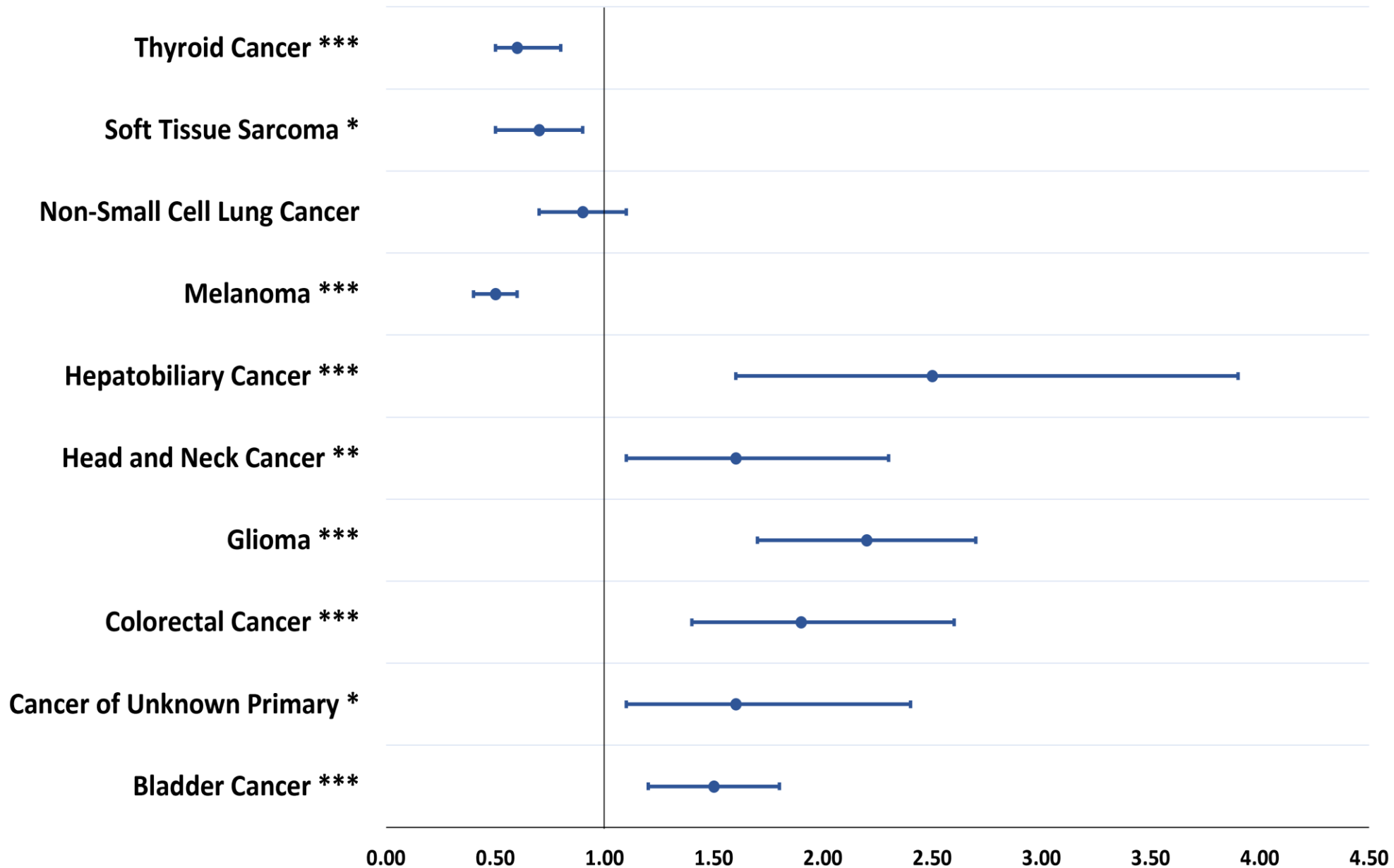

Supplement: oyad208_suppl_Supplementary_Figure_1 [file oyad208_suppl_supplementary_figure_1.pdf]
