# Supplementary material for: TERT Promoter Mutations Frequency Across Race, Sex, and Cancer Type
Source: Oncologist. 2023 Jul 18;29(1):8–14. doi: 10.1093/oncolo/oyad208 (PMC10769781; doi:10.1093/oncolo/oyad208)
Supplement: oyad208_suppl_Supplementary_Methods [file oyad208_suppl_supplementary_methods.docx]

**Supplementary Methods**

Mutational analyses in this study were focused on *TERT* promoter mutations. Methodology for tissue collection, DNA extraction and sequencing using the DFCI-ONCOPANEL and MSK-IMPACT for DFCI and MSKCC patients respectively were previously described in detail^1-4^. Details regarding genomic profiling at DFCI and MSKCC are copied below verbatim from the AACR Project GENIE DATA Guide: <https://www.aacr.org/wp-content/uploads/2022/03/GENIE_data_guide_11.0-public.pdf>.

Dana Farber Cancer Institute (DFCI)

“DFCI uses a custom, hybridization-based capture panel (OncoPanel) to detect single nucleotide variants, small indels, copy number alterations, and structural variants from tumor-only sequencing data. Three (3) versions of the panel have been submitted to GENIE: version 1 containing 275 genes, version 2 containing 300 genes, version 3 containing 447 genes. Specimens are reviewed by a pathologist to ensure tumor cellularity of at least 20%. Tumors are sequenced to an average unique depth of coverage of approximately 200x for version 1 and 350x for version 2. Reads are aligned using BWA, flagged for duplicate read pairs using Picard Tools, and locally realigned using GATK. Sequence mutations are called using MuTect for SNVs and GATK SomaticIndelDetector for small indels. Putative germline variants are filtered out using a panel of historical normals or if present in ESP at a frequency ≥ .1%, unless the variant is also present in COSMIC. Copy number alterations are called using a custom pipeline and reported for fold-change >1. Structural rearrangements are called using BreaKmer. Testing is performed for all patients across all solid tumor types. Version 3 includes the exonic regions of 447 genes and 191 intronic regions across 60 genes targeted for rearrangement detection. 52 genes present in previous versions were retired in the v3 test.”

Memorial Sloan Kettering Cancer Center (MSKCC)

“MSK uses a custom, hybridization-based capture panel (MSK-IMPACT) to detect single nucleotide variants, small indels, copy number alterations, and structural variants from matched tumor-normal sequence data (a pool of normals is used for a small subset of samples with a missing normal). Three (3) versions of the panel have been submitted to GENIE: version 1 containing 341 genes, version 2 containing 410 genes, version 3 containing 468 genes. Specimens are reviewed by a pathologist to ensure tumor cellularity of at least 10%. Tumors are sequenced to an average unique depth of coverage of approximately 750X. Reads are aligned using BWA, flagged for duplicate read pairs using GATK, and locally realigned using 13 January 7, 2022 11.0-public ABRA. Sequence mutations are called using MuTect, VarDict, and Somatic indel detector, and reported for >5% allele frequency (novel variants) or >2% allele frequency (recurrent hotspots). Copy number alterations are called using a custom pipeline and reported for fold-change >2. Structural rearrangements are called using Delly. All somatic mutations are reported without regard to biological function. Testing is performed for patients with advanced metastatic cancer across all solid tumor types”

**References**

1. Garcia EP, Minkovsky A, Jia Y, et al. Validation of OncoPanel: A Targeted Next-Generation Sequencing Assay for the Detection of Somatic Variants in Cancer. *Arch Pathol Lab Med*. Jun 2017;141(6):751-758. doi:10.5858/arpa.2016-0527-OA

2. Sholl LM, Do K, Shivdasani P, et al. Institutional implementation of clinical tumor profiling on an unselected cancer population. *JCI Insight*. Nov 17 2016;1(19):e87062. doi:10.1172/jci.insight.87062

3. Zehir A, Benayed R, Shah RH, et al. Mutational landscape of metastatic cancer revealed from prospective clinical sequencing of 10,000 patients. *Nat Med*. Jun 2017;23(6):703-713. doi:10.1038/nm.4333

4. Zehir A, Benayed R, Shah RH, et al. Erratum: Mutational landscape of metastatic cancer revealed from prospective clinical sequencing of 10,000 patients. *Nat Med*. Aug 4 2017;23(8):1004. doi:10.1038/nm0817-1004c
